# Effects of Medicare Coverage of a “Welcome-to-Medicare” Visit on Use of Preventive Services among New Medicare Enrollees

**DOI:** 10.1155/2017/2074810

**Published:** 2017-05-04

**Authors:** Boon Peng Ng, Gail A. Jensen, Heather Fritz

**Affiliations:** ^1^Department of Economics, Wayne State University, 656 W Kirby St., 2074 FAB, Detroit, MI 48202, USA; ^2^Department of Economics and Institute of Gerontology, Wayne State University, 87 East Ferry St., Detroit, MI 48202, USA; ^3^Eugene Applebaum College of Pharmacy and Health Sciences and Institute of Gerontology, Wayne State University, 259 Mack Ave., Detroit, MI 48201, USA

## Abstract

In January 2005, Medicare began covering a one-time initial preventive physical examination (IPPE), also called a “Welcome-to-Medicare” visit, during a beneficiary's first 6 months under Part B. This paper examines the effects of offering Medicare IPPE coverage on the use of mammograms, breast self-exams, Pap smears, prostate cancer screenings, cholesterol screenings, and flu vaccines among beneficiaries new to Part B. We adopt a difference-in-difference estimator and estimate a set of multivariate logit models to quantify the effects of introducing Medicare IPPE coverage on the use of preventive services. Models are estimated separately for men and women. Data for the analysis come from the 1996–2008 Health and Retirement Study. Among both men and women, having coverage for a one-time IPPE under Medicare had no effects on the utilization of any of the preventive services listed above. In this study, we find that offering coverage for a one-time IPPE under Medicare was insufficient to spur greater use of preventive services among new Medicare beneficiaries. These findings are important and suggest that policy-makers may need to consider other approaches to increase the use of recommended preventive services.

## 1. Introduction

Expanding Medicare benefits in ways to increase older adults' utilization of preventive healthcare has been a hallmark of changes to Medicare over the last 15 years. The 2003 Medicare Modernization Act (MMA), the 2008 Medicare Improvements for Patients and Providers Act (MIPPA), and most recently, the 2010 Affordable Care Act (ACA) all contained provisions intended to enhance older adults' access to affordable preventive services. The argument to encourage greater use of preventive services is that they can or will prevent more serious illnesses that are costly to treat or potentially deadly. There is broad consensus that preventive measures and quality healthcare improve overall health [1].

One key provision of the MMA involved the introduction in 2005 of Medicare coverage for a one-time initial preventive physical examination (IPPE) during a beneficiary's first six months under Medicare Part B. An IPPE, also known as a “Welcome-to-Medicare” visit, is a visit with a physician (or a physician's designee) for the purpose of introducing the beneficiary to Medicare and what it covers, talking with the beneficiary about health promotion, early disease detection, and preventive services that can help him/her stay well, and providing actual referrals for recommended preventive services [2].

In this paper, we use data from the ongoing Health and Retirement Study (HRS) to evaluate whether the introduction of Medicare IPPE coverage in 2005 improved rates of preventive services use among beneficiaries new to Part B. We examine the use of flu vaccines and cholesterol screenings among men and women, prostate cancer screenings among men, and mammograms, breast self-exams, and Pap smears among women. Because past studies have shown gender differences in health-related behaviors and medical care utilization, we examine the use of flu vaccines and cholesterol screenings separately for men and women.

We are aware of one previous study of the effects of covering an IPPE on the use of mammography and Pap smears among women aged 65 and 66 and new to Medicare Part B. Salloum and colleagues found that the use of mammograms and Pap smears among new female beneficiaries did not change following the introduction of IPPE coverage [3]. Our study examines the effects of IPPE coverage on the use of six different preventive services, including flu immunizations and five disease screening procedures, and examines these effects separately for men and women. Given that fewer than half of seniors are up-to-date on recommended preventive services [4], and expanding the use of recommended preventive services continues to be a national priority [5], the effectiveness of covering an IPPE under Medicare remains an interesting issue.

Since an IPPE is an opportunity for patients and physicians to discuss the need to use preventive services and to develop a preventive care plan, Medicare's introduction of coverage for it in 2005 may have increased the use of preventive care services among those eligible for the benefit. In this paper, we examine whether the introduction of IPPE coverage influenced the use of flu shots, cholesterol checks, and five types of cancer screening among men and women newly insured under Part B.

## 2. Methods

We analyze data from the Health and Retirement Study (HRS) and the RAND HRS. These data were chosen because the longitudinal nature of the HRS allows us to account for the effects of an individual's past utilization patterns on their current use of services, separate from the effects of Medicare IPPE coverage. (Cross-sectional data sources, such as the Medicare Current Beneficiary Survey, the Medical Expenditure Panel Survey, and the Behavioral Risk Factor Surveillance System, all lack information on individuals' past behavior regarding preventive services.) In addition, HRS data have not been used before to evaluate the effects of introducing IPPE coverage, so they may shed new light on this topic.

The HRS is a nationally representative sample survey of older adults in the US that has been conducted every two years since 1992. The survey contains copious self-reported information on health, healthcare use, insurance coverage, and sociodemographic information. The HRS first surveyed a sample of adults aged 51–61 in 1992, and this sample is called the “original HRS cohort.” A second survey, conducted in 1993 and called the Study of Assets and Health Dynamics among the Oldest Old (AHEAD), was a survey of individuals aged 70 and older. As with the HRS, spouses were also surveyed in AHEAD [7, 8]. Participants in both surveys were reinterviewed every two years, and in 1998 these two surveys were combined and have since been referred to simply as the HRS. (More information is available on the HRS website, http://hrsonline.isr.umich.edu/.)

The RAND HRS is derived from the HRS and contains many (but not all) key variables from the HRS. RAND HRS files are constructed for ease of use, and variables in the file are named and formatted to be consistent across HRS waves [6, 9].

### 2.1. Sampling Criteria

Data are drawn from the 1996, 2000, 2004, and 2008 waves (or odd number waves) of the HRS. Information on the use of preventive services is available for all HRS participants only in these waves [10]. As noted earlier, as of January 1, 2005, Medicare began covering an IPPE for beneficiaries but only during their first six months under Part B. Since most people enter Medicare when they turn 65, their eligibility for a covered IPPE would have occurred during six months when they were 65 years old. We restrict our focus to Medicare beneficiaries, aged 66–69 at the time of an HRS interview, who were insured under Part B, who were not also enrolled in Medicaid, and who were not enrolled in an HMO. We exclude beneficiaries with Medicaid because, during the period we examine (1996–2008), Medicaid already offered an ongoing preventive screening benefit, and we exclude beneficiaries with HMO coverage (Medicare Part C) because coverage of an IPPE did not apply to them; rather it was a benefit under Medicare Part B.

We divide this sample into two groups: a “treatment group” of Medicare beneficiaries aged 66 or 67 at the time of an HRS interview and a “comparison group” of Medicare beneficiaries aged 68 or 69 at the time of an HRS interview. The treatment group consists of beneficiaries aged 66 or 67 because for these individuals, at least from 2005 onward, HRS questions regarding preventive service use would have captured their six-month eligibility window for IPPE coverage. The comparison group consists of beneficiaries aged 68 or 69 because, for these individuals, HRS questions covered a two-year period well past their eligibility window for IPPE coverage.

Given our sampling criteria, each observation in our analytic sample is a distinct HRS participant and no individual contributes multiple observations across waves. Our final sample sizes by type of services are as follows:

- Mammograms: treatment group of 325 women, comparison group of 1,036 women
- Breast self-exams: treatment group of 326 women, comparison group of 1,037 women
- Pap smears: treatment group of 327 women, comparison group of 1,030 women
- Prostate cancer screenings: treatment group of 249 men, comparison group of 783 men

For cholesterol tests and flu vaccines, models are estimated separately for men and women, with sample sizes as follows:

- Cholesterol testing women: treatment group of 326, comparison group of 1,029
- Cholesterol testing men: treatment group of 254, comparison group of 784
- Flu vaccine women: treatment group of 323, comparison group of 1,031
- Flu vaccine men: treatment group of 254, comparison group of 786

### 2.2. Data Analysis

Since all of the preventive services here are binary variables, we estimate a multivariate logit model for each one and use a different-in-differences (DID) methodology to estimate the effects of introducing Medicare coverage for an IPPE [11]. The general form of the model to be estimated is

(1) logitprYi=1 ∣ X=β0+β1 Post 2005+β2 Treatment+β3 Post 2005·Treatment+β4Xi+εi,

where *Y*_*i*_ is a binary indicator for the occurrence of screening (1 if yes, 0 if no), Post 2005 indicates whether the individual's interview occurred when Medicare covered an IPPE (1 if yes, 0 if no), Treatment is a binary variable indicating membership in the treatment group (1 if yes, 0 if no), Post 2005 · Treatment is the interaction term between Post 2005 and Treatment, *X*_*i*_ is a vector of other covariates in the model, and *ε*_*i*_ is a random error term. The coefficient on the interaction term, Post 2005 · Treatment, (*β*_3_) quantifies the effect of eligibility for IPPE coverage on use of the preventive service. This estimation strategy essentially computes a difference-in-differences estimate of the effect of a beneficiary having IPPE coverage [12]. All statistical analyses were conducted in 2015 using Stata, Version 11.2 from StataCrop in College Station, Texas.

Variables in *X*_*i*_ include predisposing, enabling, and need-related variables suggested by Andersen's behavioral model of factors affecting the use of preventive healthcare [13, 14]. Predisposing factors include demographic characteristics, social structure, and health beliefs. Enabling factors encompass the economic accessibility of services (e.g., insurance and income), as well as the availability of services nearby [15, 16]. Finally, need factors account for an individual's current state of health, as well as their attitudes about their needs for healthcare based on how they perceive their own health [17]. Although past experience with preventive services is not explicit in Andersen's model, we think it may reflect an individual's attitudes regarding the value of such services and may also reflect their past health habits. As such, it belongs to behavioral models of the use of preventive services.

Andersen's model was adapted by Walsh and McPhee (1992) to account for the potential effects of patient-physician interactions on the use of services. In their “Systems Model of Clinical Preventive Care” Walsh and McPhee posit that seven categories of factors affect the use of preventive healthcare services: predisposing factors, enabling factors, reinforcing factors, healthcare delivery system factors, situational factors (cues to action), and preventive activity factors [18]. Situational factors are particular circumstances or cues that may trigger the use of preventive services. An example is a patient receiving a physician's recommendation or a reminder to use preventive services [19]. Past studies have shown that recommendations from physicians are a strong predictor of use of preventive services among older adults, and patients who have discussions about preventive services with their physician are much more likely to use such services [20–22].

Our predisposing variables include marital status, race, education, and whether the individual previously used that particular preventive service. Enabling-related variables include access to additional insurance beyond Medicare, such as an employer-sponsored policy or a Medigap plan, income, region of residence, urban/rural location, employment status, and whether the individual is able to drive. Need-related variables in each model include whether the individual smokes, drinking habits, whether the individual is overweight, the presence of chronic diseases, self-rated health, eyesight, exercise habits, performance on activities of daily living (ADL), and mental health, as measured by the Center for Epidemiologic Studies Depression Scale (CES-D).

## 3. Results

Table 1 reports definitions and descriptive statistics for variables used in this analysis. During the preperiod, that is, before Medicare covered an IPPE, 76% of women in the treatment group and 80% in the comparison group reported receiving a mammogram during the past two years. Sixty-five percent of women in the treatment group and 61% in the comparison group reported they were checking their breasts for lumps monthly. Sixty-four percent of women in the treatment group and 63% in the comparison group reported receiving a Pap smear during the past two years. Seventy-eight percent of men in the treatment group and 81% in the comparison group reported receiving a prostate exam during the past two years. Sixty-eight percent of men in the treatment group and 67% in the comparison group reported receiving a flu vaccine during the past two years. Sixty-seven percent of women in the treatment group and 70% in the comparison group reported receiving a flu vaccine during the past two years. Eighty-seven percent of men in the treatment group and 84% in the comparison group reported receiving a cholesterol test. Finally, 82 percent of women in the treatment group and 84% in the comparison group reported receiving a cholesterol test during the past two years.

Tables 2 and 3 report the odds ratios from the estimated logit regressions for the use of various preventive services. For each model the odds ratio of key interest is the one corresponding to the row labeled “Post 2005 *∗* Treatment.” For all six preventive services, the estimated coefficient of policy effect indicator is statistically insignificant. This implies that having a six-month window of Medicare coverage for a one-time IPPE had no effects on the use of mammograms, breast self-exams, Pap smears, prostate cancer screenings, cholesterol tests, or flu vaccines among new Medicare enrollees.

Several other factors, however, were highly predictive of preventive services utilization, and we briefly discuss them here. Among women, those who previously received a mammogram were 10.81 times more likely to have one again (Table 2). Having employer-provided insurance (in addition to Medicare) increased a woman's likelihood of having a mammogram by 1.56. Full-time employment, nondrinkers, and the absence of any chronic diseases reduced the likelihood of having a mammogram by 0.48, 0.68, and 0.55 times, respectively. Women who were nonsmokers were 2.15 times more likely to receive a mammogram.

For breast self-exams (Table 2), women who had previously checked their breasts for lumps were 13.88 times more likely to check them again. Women living in a rural area, who were employed, or who were married were more likely to check for breast lumps; and those with only a high school education or GED were less likely to perform a breast self-exam.

Women who previously received a Pap smear (Table 2) were 7.50 times more likely to receive another one. Having employer-provided insurance also improved the odds of receiving a Pap smear.

Men who previously received a prostate exam (Table 2) were 4.75 times more likely to receive another one. Men who were more highly educated were also more likely to be screened. Nonsmokers and men who scored zero on the CES-D were also more likely to be screened. Having no chronic diseases and having no ADL limitations had negative effects on receiving a prostate exam.

For cholesterol tests (Table 3), women who previously had a cholesterol test were 5.11 times more likely to have another one, whereas men who previously had the test were 7.73 times more likely to have another one. Both women and men who do not smoke and who exercised regularly were more likely to have their cholesterol checked. In contrast, women and men without chronic diseases were less likely to be tested for cholesterol levels. Men with less-than-good health, with some college education and beyond, who were currently married, who were able to drive, who do not drink, and who scored zero on the CES-D were more likely to take a cholesterol test. Men having no ADL limitations were less likely to have had a cholesterol test. Finally, higher income had a positive effect on the use of cholesterol tests, but only among women.

Women and men who previously received a flu vaccine (Table 3) were 17.57 and 16.55 times more likely to receive another one, respectively. Women and men who were nonsmokers, and who had at least some college education were more also likely to receive a flu vaccine. However, among both women and men, those with no chronic diseases were less likely to receive one. Hispanic women were less likely to be vaccinated against flu, compared to both (non-Hispanic) White or Black women. Finally, women who were married and with less-than-good health were more likely to be vaccinated.

## 4. Discussion

This analysis of data from the 1996–2008 HRS reveals that covering a one-time IPPE had no effects on the use of mammograms, breast self-exams, Pap smears, prostate cancer screenings, cholesterol tests, or flu vaccines among new Medicare enrollees. Neither men nor women changed their use of preventive services in response to the availability of IPPE coverage.

For all six preventive care services, the single strongest predictor of use was previous utilization of that service. It has not been widely used by many empirical studies and should be taken into consideration as a key factor that can affect the use of preventive services. Other factors such as having no chronic diseases, having no ADL limitations, not smoking, having supplemental health insurance, being married, being more educated, and being able to drive also affected the use of preventive services.

In our analysis of cholesterol screenings and flu vaccines, models were estimated separately for men and women. We found that the estimated coefficients of explanatory variables in these models differ for men and women. This finding, which is consistent with past studies cited, reveals that the demand for these services indeed differs by gender.

In general, the equations we estimated accord with an Andersen-type model of healthcare utilization. Our finding that an individual's prior screening habits were the strongest predictor of their current utilization, for example, suggests that reinforcing factors and overall health beliefs play a critical role when it comes to preventive care. Education, which is a predisposing factor, and having additional health insurance beyond Medicare, which is an enabling factor, were also found to be important. Our measures of need-related factors, likewise, were often predictive of actual use of services.

To ensure the robustness of our key result, namely, that covering an IPPE had no effects on the use of preventive services, several sensitivity analyses were also conducted. Yet with every robustness check the same finding of no effects emerged. Specifically, we first reestimated the models using different specifications, excluding and including key variables [11]. We also reestimated all of them without the “previous use of preventive care” as an independent variable. We then reestimated them excluding those variables that were not statistically significant. In each case, the results remained the same: the policy indicator was still statistically insignificant. We also reestimated the model only using data from waves 7 (year 2004) and 9 (year 2008), to provide evenly balanced sample counts across the pre- and postperiods. Yet, in this case too, the policy indicator remained insignificant, except for prostate cancer screenings, where it showed a positive effect on receiving a prostate cancer screening. The models were also reestimated using an alternative comparison group of individuals aged 72 and 73 (not affected by the policy change), and in these models the coefficient on the policy indicator remained insignificant. The results therefore suggest that the use of preventive services by new Medicare enrollees was not affected by coverage of an IPPE.

There are likely multiple reasons for why beneficiaries have not fully utilized the IPPE benefit. Some beneficiaries may have been simply unaware of Medicare's coverage of an IPPE, or they may have first learned about it too late to take advantage of it. Recall when the IPPE benefit was first introduced in 2005 it was only available during a beneficiary's first six months under Medicare. For example, if someone learned about it four or five months after enrollment, they may not have been able to schedule an IPPE inside their six-month window. Another explanation is that some people simply do not assign high value to this sort of visit. It is unrealistic, for example, to think that all individuals would suddenly prioritize preventive services if they had not been doing so in the past, simply because a cost barrier had been removed. Moreover, although the IPPE benefit is “free,” beneficiaries still must often coordinate transportation and expend the time and energy to attend the appointment.

Time, motivation, and awareness aside, there are also logistic issues that may have prevented some providers from fully embracing the IPPE. On the provider side one concern is that an IPPE visit is supposed to be separate from other routine primary care procedures. In essence, Medicare intends for the IPPE to focus on discussing health promotion and disease prevention. If, in the middle of an IPPE, a patient asks the physician to look at a rash or examine a sore throat, addressing this violates the intent of an IPPE. Yet, patients are not accustomed to going to the doctor to* not* discuss problems, and, understandably, physicians may be reticent to tell a patient who is already in front of them that they should make another appointment to discuss their sore throat. While it is possible for a physician to add a second service code to an IPPE visit claim with a modifier that enables payment for both services, the modifier pays less than a separate evaluation and management visit would pay. This creates a reimbursement dilemma for the physician: should they accept lower payment and address the patient's ailment then-and-there or suggest the patient make another appointment? Complicating the issue is that many physicians simply do not have extra time available to squeeze in an additional same-day visit for an IPPE patient, and even if they could, that patient might be unable or unwilling to stay and wait. In short, after spending the time and energy to attend a primary care appointment many, if not all, patients expect to be able to discuss any issue with their physician that is relevant at the time and not to have to make second appointments.

Another complication arises from patients' likely confusion over what can and cannot be done during the IPPE. Many patients liken their annual physical to a health promotion and prevention visit and, therefore, may not understand why a second visit, namely, the IPPE, is necessary. This may be especially confusing for a patient because the physician does not have them undress, check their vitals, or conduct a physical exam during the IPPE. Furthermore, some physicians may have already been combining the principles of the IPPE in their patients' routine physicals and since Medicare does not cover physicals, they do not submit a claim for the IPPE.

The various explanations discussed above for why the IPPE has not been embraced under Medicare are largely speculative on our part. As far as we know, empirical studies have not yet been conducted to pin down the reasons for the low utilization of IPPEs.

This study has two limitations which should be noted. The HRS asked participants about their use of preventive services over the past two years. We would have preferred it to have asked “over the last year,” as this would have allowed us to capture the effects of IPPE coverage more accurately. Some might question the validity of using self-reported healthcare utilization data, especially in a sample of older adults.

## 5. Conclusions

Efforts to improve the use of preventive services are important as the aging population increases in the US, and as quality healthcare, including preventive care, becomes imperative. In addition, fewer than half of seniors are up-to-date on recommended preventive services [4]. Despite this need, the findings presented here strongly suggest that, at least as it was initially designed, covering an IPPE under Medicare had no significant effects on the use of preventive services, suggesting that time-limiting benefits or health policies may not provide desired public health outcomes. Policy-makers should be aware of this limitation and consider other approaches to increase the use of preventive services under Medicare.

## Figures and Tables

**Table 1:** Variable definitions and descriptive statistics for the treatment and comparison groups before Medicare introduced IPPE coverage, Health and Retirement Study, 1996–2004.

Variable	Definition	Treatment Group		Comparison Group	
		Mean (SE)		Mean (SE)	
*Dependent variables*					
Mammogram^a^	1 if having a mammogram; 0 otherwise	0.76 (0.42)		0.80 (0.39)	
Check for breast lumps^a^	1 if conducting a monthly self-exam for breast lumps; 0 otherwise	0.65 (0.47)		0.61 (0.48)	
Pap smear^a^	1 if having a Pap smear; 0 otherwise	0.64 (0.47)		0.63 (0.48)	
Prostate exam^a^	1 if having a prostate examination; 0 otherwise	0.78 (0.41)		0.81 (0.38)	
		Men	Women	Men	Women
Cholesterol test^a^	1 if having a cholesterol blood test; 0 otherwise	0.87 (0.33)	0.82 (0.38)	0.84 (0.36)	0.84 (0.36)
Flu vaccine^a^	1 if having a flu shot; 0 otherwise	0.68 (0.46)	0.67 (0.47)	0.67 (0.46)	0.70 (0.45)
*Control variables*					
Previous use of mammogram^a^	1 if previously having a mammogram; 0 otherwise	0.74 (0.43)		0.77 (0.41)	
Previous breast self-exam^a^	1 if previously conducting a monthly self-exam for breast lumps; 0 otherwise	0.62 (0.48)		0.63 (0.48)	
Previous use of Pap smear^a^	1 if previously having a Pap smear; 0 otherwise	0.67 (0.47)		0.70 (0.45)	
Previous use of prostate exam^a^	1 if previously having a prostate examination; 0 otherwise	0.76 (0.42)		0.75 (0.43)	
		Men	Women	Men	Women
Previous use of cholesterol test^a^	1 if previously having a cholesterol blood test; 0 otherwise	0.74 (0.43)	0.74 (0.43)	0.77 (0.41)	0.79 (0.40)
Previous use of flu vaccine shot^a^	1 if previously having a flu shot; 0 otherwise	0.51 (0.50)	0.53 (0.49)	0.49 (0.50)	0.54 (0.49)
Race					
White	1 if White/Caucasian; 0 otherwise	0.76 (0.42)		0.83 (0.37)	
Black	1 if Black/African American; 0 otherwise	0.14 (0.35)		0.11 (0.32)	
Hispanic	1 if Hispanic/Latino; 0 otherwise	0.06 (0.25)		0.03 (0.18)	
Other	1 if a race/ethnicity other than White, Black, or Hispanic; 0 otherwise	0.08 (0.28)		0.05 (0.22)	
Education					
Less than high school	1 if less than 12 years of education; 0 otherwise	0.22 (0.41)		0.17 (0.37)	
High school/GED	1 if 12 years of education; 0 otherwise	0.41 (0.49)		0.41 (0.49)	
Some college and beyond	1 if more than 12 years of education; otherwise	0.36 (0.48)		0.41 (0.49)	
Employment	1 if employed full time; 0 otherwise	0.09 (0.28)		0.08 (0.28)	
CES-D score^b^	1 if scored 0 in CESD; 0 otherwise	0.42 (0.49)		0.51 (0.50)	
Chronic diseases	1 if reporting 0 chronic diseases; 0 otherwise	0.13 (0.34)		0.13 (0.34)	
Married	I if married; 0 otherwise	0.71 (0.45)		0.73 (0.44)	
Census region					
Northeast	1 if census region is northeast; 0 otherwise	0.13 (0.34)		0.11 (0.32)	
Midwest	1 if census region is midwest; 0 otherwise	0.28 (0.45)		0.32 (0.46)	
South	1 if census region is south; 0 otherwise	0.44 (0.49)		0.44 (0.49)	
West	1 if census region is west; 0 otherwise	0.13 (0.34)		0.11 (0.32)	
Rural	1 if population is less than 250,000; 0 otherwise	0.39 (0.48)		0.38 (0.48)	
Exercise	1 if performing physical activity; 0 otherwise	0.74 (0.43)		0.75 (0.42)	
Not drinking	1 if not drinking; 0 otherwise	0.71 (0.45)		0.67 (0.46)	
Not smoking	1 if not smoking; 0 otherwise	0.82 (0.37)		0.87 (0.32)	
Driving	1 if being able to drive; 0 otherwise	0.90 (0.29)		0.94 (0.23)	
Total household real income (in year 2007 dollars)					
Income 1	1 if total household income is less than $25000; 0 otherwise	0.28 (0.45)		0.23 (0.42)	
Income 2	1 if total household income is between $25,000 and $50,000; 0 otherwise	0.32 (0.46)		0.34 (0.47)	
Income 3	1 if total household income is more than $50,000, 0 otherwise	0.39 (0.48)		0.42 (0.49)	
Employer provided insurance	1 if covered by employer insurance; 0 otherwise	0.43 (0.490		0.44 (0.49)	
ADL^c^	1 if reporting 0 ADL limitations; 0 otherwise	0.86 (0.34)		0.90 (0.29)	
Overweight	1 if BMI is equal to and greater than 25; 0 otherwise	0.70 (0.45)		0.68 (0.46)	
Self-reported health					
Better than good	1 if reporting better than good health; 0 otherwise	0.36 (0.48)		0.48 (0.50)	
Good	1 if reporting good health; 0 otherwise	0.30 (0.46)		0.31 (0.46)	
Less than good	1 if reporting worse than good health; 0 otherwise	0.32 (0.46)		0.20 (0.40)	
Rate eyesight					
Better than good	1 if reporting better than good eyesight; 0 otherwise	0.32 (0.47)		0.39 (0.48)	
Good	1 if reporting good eyesight; 0 otherwise	0.45 (0.49)		0.44 (0.49)	
Less than Good	1 if reporting worse than good eyesight; 0 otherwise	0.22 (0.41)		0.15 (0.36)	

*Note*. SE = standard error.

^a^Among a specific preventive care group only.

^b^Center for Epidemiologic Studies Depression Scale (CES-D) is the sum of indicators: feeling depressed, everything being an effort, sleep being restless, feeling unhappy, feeling lonely, feeling sad, could not get going, and not enjoying life [6].

^c^The activities of daily living (ADL) index covers walking across a room, dressing, bathing, eating, getting in and out of bed, and using the toilet [6].

**Table 2:** Odds ratios derived from multivariate logit models for having a mammogram, breast self-exam, Pap smear, or prostate cancer screening (Health and Retirement Study data, 1996–2008).

	Mammogram	Breast self-exam	Pap smear	Prostate
	Odds ratio (95% CI)	Odds ratio (95% CI)	Odds ratio (95% CI)	Odds ratio (95% CI)
Post 2005	0.94 (0.62–1.41)	0.97 (0.69–1.36)	0.75^a^ (0.55–1.03)	0.64^b^ (0.42–0.97)
Treatment	0.87 (0.55–1.37)	1.33 (0.89–1.99)	1.23 (0.84–1.80)	0.85 (0.51–1.40)
Post 2005*∗*Treatment	0.89 (0.42–1.88)	0.85 (0.43–1.65)	1.19 (0.64–2.21)	1.70 (0.74–3.89)
Previous use of the service	10.81^c^ (7.80–14.99)	13.88^c^ (10.52–18.32)	7.50^c^ (5.70–9.88)	4.75^c^ (3.29–6.86)
Married	1.13 (0.78–1.65)	1.50^b^ (1.08–2.09)	1.19 (0.88–1.62)	1.13 (0.71–1.81)
White	0.36 (0.09–1.40)	0.88 (0.29–2.64)	0.55 (0.20–1.46)	0.64 (0.15–2.67)
Black	0.65 (0.15–2.75)	0.75 (0.24–2.39)	0.87 (0.30–2.44)	0.92 (0.20–4.12)
Hispanic	0.34 (0.07–1.50)	0.45 (0.13–1.53)	0.62 (0.20–1.90)	0.67 (0.13–3.31)
High school/GED	0.99 (0.64–1.53)	0.64^b^ (0.43–0.96)	1.10 (0.77–1.59)	1.58^a^ (0.98–2.55)
Some college and beyond	1.12 (0.69–1.80)	0.81 (0.53–1.24)	1.16 (0.79–1.70)	1.65^a^ (0.98–2.76)
Employer provided insurance	1.56^b^ (1.10–2.22)	0.89 (0.67–1.18)	1.34^b^ (1.02–1.76)	1.12 (0.77–1.62)
Employment	0.48^b^ (0.27–0.86)	1.82^b^ (1.03–3.23)	0.94 (0.57–1.54)	1.30 (0.76–2.24)
Driving	1.53 (0.91–2.57)	0.95 (0.58–1.58)	0.71 (0.45–1.12)	2.27 (0.72–7.09)
Income 2	1.30 (0.85–1.99)	1.08 (0.73–1.57)	1.24 (0.87–1.76)	1.00 (0.61–1.63)
Income 3	1.25 (0.77–2.02)	0.95 (0.62–1.44)	1.34 (0.90–1.98)	1.27 (0.74–2.19)
Northeast	1.02 (0.54–1.92)	1.21 (0.70–2.10)	1.65^a^ (0.99–2.76)	0.61 (0.30–1.25)
Midwest	0.79 (0.46–1.35)	0.71 (0.45–1.12)	1.06 (0.69–1.62)	0.74 (0.40–1.36)
South	1.20 (0.72–2.02)	0.86 (0.56–1.34)	1.12 (0.74–1.68)	0.83 (0.46–1.47)
Rural	1.18 (0.84–1.67)	1.33^a^ (0.99–1.78)	0.97 (0.74–1.28)	1.07 (0.74–1.55)
Not smoking	2.15^c^ (1.40–3.30)	1.24 (0.82–1.86)	1.11 (0.76–1.63)	2.17^c^ (1.37–3.43)
Not drinking	0.68^a^ (0.45–1.03)	1.05 (0.75–1.46)	0.79 (0.57–1.09)	1.09 (0.76–1.56)
Overweight	0.84 (0.59–1.21)	1.03 (0.76–1.39)	0.97 (0.72–1.29)	1.03 (0.69–1.53)
Exercise	1.19 (0.79–1.79)	0.86 (0.60–1.25)	1.17 (0.83–1.66)	1.25 (0.76–2.04)
No chronic diseases	0.55^b^ (0.34–0.89)	1.17 (0.75–1.81)	0.88 (0.58–1.33)	0.46^c^ (0.28–0.76)
No ADL	1.38 (0.85–2.24)	0.79 (0.50–1.26)	1.25 (0.82–1.89)	0.55^a^ (0.30–1.02)
Zero CES-D	1.30 (0.91–1.84)	0.88 (0.65–1.18)	1.11 (0.84–1.47)	1.64^c^ (1.13–2.39)
Better than good health	0.73 (0.48–1.10)	0.85 (0.60–1.19)	1.03 (0.75–1.42)	0.93 (0.61–1.43)
Less than good health	0.84 (0.54–1.32)	0.90 (0.60–1.34)	0.91 (0.63–1.31)	1.49 (0.91–2.44)
Better than good eyesight	1.47^b^ (1.01–2.14)	0.80 (0.59–1.10)	1.29^a^ (0.96–1.73)	1.42^a^ (0.94–2.15)
Less than good eyesight	1.12 (0.71–1.76)	0.97 (0.65–1.46)	1.02 (0.70–1.48)	0.90 (0.55–1.45)
Pseudo *R*-squared	0.25	0.26	0.17	0.16
Sample size	*N* = 1365	*N* = 1363	*N* = 1357	*N* = 1032

*Notes*. CI = confidence interval. Sample sizes vary across models because some individuals are missing data on some items and because there are different numbers of women and men in the sample.

^a^Significant at 10%, ^b^significant at 5%, and ^c^significant at 1%.

**Table 3:** Odds ratios derived from multivariate logit models for the receipt of cholesterol testing and flu vaccine (Health and Retirement Study data, 1996–2008).

	Cholesterol testing		Flu vaccine	
	Women	Men	Women	Men
	Odds ratio (95% CI)	Odds ratio (95% CI)	Odds ratio (95% CI)	Odds ratio (95% CI)
Post 2005	1.40 (0.88–2.25)	1.06 (0.62–1.80)	0.76 (0.52–1.12)	0.63^b^ (0.42–0.96)
Treatment	0.97 (0.60–1.55)	2.11^b^ (1.09–4.08)	0.87 (0.56–1.35)	1.08 (0.67–1.73)
Post 2005*∗*Treatment	1.19 (0.48–2.91)	0.83 (0.26–2.60)	0.85 (0.41–1.77)	0.79 (0.36–1.73)
Previous use of the service	5.11^c^ (3.57–7.32)	7.73^c^ (4.94–12.10)	17.57^c^ (12.55–24.59)	16.55^c^ (11.41–23.99)
Married	1.29 (0.84–1.95)	1.63^a^ (0.94–2.83)	1.39^a^ (0.96–2.01)	1.38 (0.87–2.20)
White	0.27 (0.05–1.55)	0.96 (0.19–4.92)	1.51 (0.50–4.55)	0.56 (0.14–2.24)
Black	0.24 (0.04–1.42)	0.84 (0.15–4.67)	0.65 (0.20–2.07)	0.39 (0.09–1.69)
Hispanic	0.26 (0.04–1.72)	0.85 (0.13–5.44)	0.32^a^ (0.09–1.16)	0.54 (0.11–2.53)
High school/GED	0.99 (0.61–1.60)	1.63 (0.90–2.94)	1.27 (0.83–1.94)	1.63^b^ (1.01–2.64)
Some college and beyond	1.40 (0.83–2.39)	2.12^b^ (1.11–4.03)	1.55^a^ (0.98–2.45)	1.79^b^ (1.07–2.98)
Employer provided insurance	1.20 (0.82–1.76)	0.88 (0.55–1.40)	1.09 (0.79–1.50)	1.05 (0.75–1.49)
Employment	0.78 (0.41–1.47)	1.14 (0.60–2.17)	0.76 (0.42–1.35)	0.88 (0.54–1.43)
Driving	0.83 (0.45–1.54)	9.28^c^ (2.57–33.43)	0.87 (0.49–1.52)	1.28 (0.32–5.09)
Income 2	1.56^a^ (0.97–2.51)	0.68 (0.36–1.28)	0.81 (0.53–1.24)	1.14 (0.70–1.87)
Income 3	1.32 (0.78–2.25)	0.65 (0.32–1.30)	0.72 (0.45–1.15)	0.84 (0.50–1.41)
Northeast	1.42 (0.66–3.04)	0.51 (0.18–1.43)	1.28 (0.67–2.41)	0.85 (0.42–1.69)
Midwest	0.91 (0.49–1.69)	0.42^a^ (0.17–1.02)	0.62^a^ (0.36–1.04)	0.70 (0.39–1.25)
South	0.79 (0.44–1.41)	0.45^a^ (0.19–1.04)	0.67 (0.40–1.11)	0.68 (0.40–1.17)
Rural	0.88 (0.61–1.27)	0.66^a^ (0.42–1.04)	1.11 (0.80–1.54)	1.06 (0.75–1.51)
Not smoking	1.44 (0.90–2.28)	1.98^b^ (1.12–3.50)	1.90^c^ (1.22–2.96)	1.62^a^ (0.99–2.64)
Not drinking	0.88 (0.57–1.36)	1.64^b^ (1.05–2.56)	0.94 (0.65–1.36)	0.92 (0.65–1.29)
Overweight	1.02 (0.69–1.49)	1.43 (0.90–2.29)	1.31 (0.93–1.83)	1.22 (0.82–1.80)
Exercise	1.76^c^ (1.14–2.69)	1.69^a^ (0.96–2.96)	1.20 (0.80–1.81)	0.98 (0.61–1.58)
No chronic diseases	0.36^c^ (0.23–0.57)	0.30^c^ (0.17–0.53)	0.63^b^ (0.40–0.99)	0.64^a^ (0.39–1.03)
No ADL	1.22 (0.68–2.20)	0.45^a^ (0.18–1.09)	0.93 (0.55–1.54)	1.01 (0.55–1.88)
Zero CES-D	0.86 (0.58–1.25)	2.01^c^ (1.25–3.22)	1.07 (0.77–1.48)	1.00 (0.70–1.43)
Better than good health	0.69 (0.44–1.07)	0.94 (0.55–1.60)	1.17 (0.80–1.70)	0.81 (0.54–1.21)
Less than good health	0.97 (0.57–1.64)	2.46^c^ (1.28–4.70)	1.99^c^ (1.26–3.14)	1.42 (0.87–2.29)
Better than good eyesight	0.95 (0.64–1.42)	1.22 (0.73–2.03)	1.06 (0.75–1.50)	1.43^a^ (0.98–2.10)
Less than good eyesight	0.98 (0.58–1.64)	0.59^a^ (0.32–1.07)	1.01 (0.66–1.57)	1.00 (0.62–1.60)
Pseudo *R*-squared	0.16	0.26	0.32	0.28
Sample size	*N* = 1355	*N* = 1038	*N* = 1354	*N* = 1040

*Notes*. CI = confidence interval. Sample sizes vary across models because some individuals are missing data on some items and because there are different numbers of women and men in the sample.

^a^Significant at 10%, ^b^significant at 5%, and ^c^significant at 1%.
